# Safety During Ureteroscopy: Radiation, Eyes, and Ergonomics

**DOI:** 10.3389/fsurg.2021.737337

**Published:** 2021-10-28

**Authors:** David T. Miller, Michelle J. Semins

**Affiliations:** Department of Urology, University of Pittsburgh Medical Center, Pittsburgh, PA, United States

**Keywords:** endourologic surgery, surgical ergonomics, radiation safety, ureteroscopy (URS), nephrolithiasis

## Abstract

It is known that urologic surgeons are at risk of work-place injury due to the physical requirements of operating and exposure to hazards. These hazards include radiation, exposure to body fluids, use of laser energy, and orthopedic injury due to the physical nature of operating. The risks that these hazards present can be mitigated by implementing several evidence-based safety measures. The methods to protect against radiation exposure include keeping radiation usage in the operating room as low as reasonably achievable, donning lead aprons, and wearing protective glasses. Additionally, protective glasses decrease the risk of eye injury from laser injury and exposure to body fluids. Finally, practicing sound surgical ergonomics is essential to minimize the risk of orthopedic injury and promote career longevity. The interventions discussed herein are simple and easy to implement in one's daily practice of urology.

## Introduction

Safety in the operating room is of paramount importance to the patient, but also to the surgeon and staff in the room. Here we discuss evidence-based solutions to minimize risk to the surgeon and operating room staff. We concentrate on the areas of minimizing radiation exposure, use of eye protection, and practicing sound ergonomics to improve safety in the operating room.

## Reducing Radiation Exposure

Radiation mitigation is at the forefront of efforts to improve both patient and medical staff safety. The use of radiation to aid in the diagnosis, treatment, and follow up of stone disease is, for now, unavoidable. Endourologists who regularly perform fluoroscopically-guided procedures are at risk for higher levels of radiation exposure.

Radiation has side effects on the human body and are divided into 2 categories: stochastic and deterministic. Deterministic effects occur after an acute exposure over a specific threshold of radiation dosage. Examples include hair loss, cataracts and dermal burns (1). These effects are not typically seen by the urologic surgeon as these radiation thresholds are not reached in the treatment of stone disease. Of more relevance to the urologist is the risk of secondary malignancy due to radiation exposure, which is a stochastic effect. “Stochastic” meaning that it occurs in a linear fashion with dose, age, and gender-dependent factors playing a role. The evidence for this mechanism is derived from studies reporting on increased risk of secondary malignancy seen in patients with exposure to nuclear explosions, nuclear powerplant workers, and patients with conditions requiring repeated computed tomography (CT) scans (2–5).

The stochastic effects of radiation exposure are of significance particularly for the operating urologist. Traditionally, ureteroscopy has always been fluoroscopically guided. The maximum 1-year allowable radiation dosage is 50 or 20 mSv per year over a 5-year period per International Commission on Radiologic Protection (ICRP) Occupational guidelines (1). Fortunately, there is no existing evidence of increased risk of secondary malignancy in urologists due to occupational radiation exposure. There has been historical evidence of increased risk of leukemia RR 3.86 (1.21–12.3) among interventional cardiologists and radiologists who graduated medical school before 1940, but these authors did not find an increased risk of mortality in physicians who graduated in the following decades (6). This is likely due, in part, to a continued decrease in the estimated annual radiation exposure for radiation technologists from 710 mSv in the 1930s down to 5.5 mSv in the 1990s (6). There have been 2 studies that have recently quantified this exposure for percutaneous nephrolithotomy (PCNL) and they report that the mean exposure by operating surgeons is around 0.05–0.21 mSv per case, as measured by dosimeters worn within lead aprons (7). This is notably well within safety parameters. Overall, it is difficult to accurately report exposure because studies often extrapolate data from a single or small number of surgeons with varying experiences. With significant practice volume and case complexity variation, generalizability is challenging. However, while dosage per case can be low, additional exposure received over the course of a career may add up. The Committee on the Biological Effects of Ionizing Radiation reports that, if following the linear-no-threshold theory, a dose of about 37.3 mSv carries a lifetime attributable risk of secondary malignancy ranging from 0.40% in young females to 0.065% in older men (8).

Easy to implement interventions have been shown to reduce fluoroscopy time by up to 80%. These include radiation safety training, wearing dosimeters, and instituting formal radiation reduction protocols with pre-operative checklists (9–12). Further techniques to decrease fluoroscopy time include using radiology technicians familiar with urologic procedures, collimation, using the C-arm laser beam to target organ location without image exposure, using markings on the drape to guide the laser to the organ of interest, and using last image hold functions to avoid unnecessary duplicate fluoroscopic image acquisition (13). Also, setting the C-arm to low dose and using pulsed fluoroscopy has resulted in significantly decreased radiation dosages. Switching to the low dose setting has been shown to decrease radiation dosage per case by 57% (14). Limiting pulses to 4 frames per second has decreased total fluoroscopy time for ureteroscopy from 109.1 to 44.1 s (*P* < 0.001) (15). Even using 1 frame per second is feasible and results in significantly decreased radiation exposure (16). Unsurprisingly, switching to lower frames per second also reduced surgeon radiation dosimeter measurements by 60% (17). In addition, we encourage foot pedal control by the surgeon, establishing a common terminology with the radiation technologist pre-operatively, and having the technologist mark the floor to demonstrate the appropriate C-arm position for bladder and kidney images. We find these steps minimize fluoroscopy usage when transitioning between kidney and bladder images and facilitates seamless turnover between technologists during the case.

Steps have been taken to further eliminate fluoroscopy in ureteroscopy altogether. A group from Turkey published their outcomes performing retrograde ureteroscopy without the use of fluoroscopy, instead utilizing semi-rigid ureteroscopy to verify access to the renal pelvis. They found that without fluoroscopy there was no difference in operative times, complications, or stone-free rates. These authors advocate eliminating the use of fluoroscopy especially in cases when accessing the renal pelvis can first be achieved with direct visualization (18). Ultrasonography has also been shown to be efficacious to help guide ureteroscopy. Deters et al. performed a randomized controlled trial comparing use of fluoroscopic vs. ultrasound guided ureteroscopy. They reported no difference in complication or stone free rates between the 2 groups (19). Additionally, Olgin et al. conducted a small, randomized controlled trial comparing the outcomes of fluoro-less ureteroscopy with the use of fluoroscopy, and found that in non-complex cases, forgoing the use of fluoroscopy was safe and efficacious (20).

Lastly, wearing protective equipment such as lead aprons, thyroid shields, and lead glasses is the most obvious way to protect oneself from radiation exposure. However, numerous studies have shown surprisingly variable and, in some cases, very poor compliance with shielding. As few as 50% of surgeons wear thyroid shields in some surveys and no survey has shown higher than 50% of lead eyewear use. Also worrisome is that most endourologists do not even wear a dosimeter to track their exposure to ensure they are within safe exposure ranges (21–23).

## Protecting Your Eyes

The eye is a key organ to protect with 3 potential risks during ureteroscopy. First, the eye is especially radiosensitive with one of the known deterministic effects of radiation being the formation of cataracts. Second, the use of laser energy could cause injury. And, finally, there is risk to the eye of exposure to bodily fluids. In a large review of the existing literature Doizi et al. reported that surgeon eye lens radiation dose ranged from 2.97 to 100 uSv per ureteroscopy. The reported long-term doses of radiation that lead to cataract formation range from 2,500 to 6,500 mSv (24). Over the entirety of a career assuming a mean dose of 0.208 mSv per case, averaging 20 cases per month, it would take about 50 years to reach the minimum threshold for cataract formation (25). Thus, the threshold for cataract formation is not likely to be reached over the career of the general urologist. However, high volume endourologists could be at risk. Lead glasses reduce this exposure by up to 95% and, also protect against other exposures which will be subsequently discussed (26).

Second, the safety of the use of laser energy to treat urolithiasis has been looked at extensively. Althunayan et al. report that eye injuries account for 37.9% of all adverse events related to laser usage. It should be noted that the degree of these injuries ranges from mild corneal abrasions to complete vision loss, with no reported eye injuries when eye protection was worn (27). Villa et al. studied the effect of holmium laser on eyes using an *ex vivo* animal model on pig eyes with various laser settings. The authors reported no injuries >5 cm from cornea (regardless of settings and time of lasering), and no injuries with laser safety glasses or with regular eyeglasses. They concluded regular eyeglasses are as effective as laser safety glasses for protecting eyes from holmium laser exposures (28). There are no reported eye injuries with holmium or thulium lasers. However, it is recommended to wear eye protection to cover the adequate wavelength with neodymium-doped yttrium aluminum garnet (Nd:YAG), potassium titanyl phosphate (KTP), and diode lasers as there has been reported eye injuries with these modalities due to their depth of tissue penetration and shorter wavelength (Table 1) (27). Lasers with shorter wavelengths, specifically near that of visible light (400–780 nm), can cause more damage such as thermal retinal injury and photokeratitis, than those with longer wavelengths (37).

**Table 1:** Depth of tissue penetration by type of laser (29–34).

**Type of laser**	**Depth of tissue penetration (mm)**	**Wavelength (nm)**
Thulium:YAG	0.5–2	1,910
Holmium:YAG	0.5–1	2,100
KTP:YAG	1	532
ND:YAG	5–6	1,064
Diode	8–9	810–830

Third, is the risk of surgeon exposure to bodily fluids. Wines et al. found that surgeon eye exposure to patient blood droplets is as high as 50% during ureteroscopy (38). Fortunately, the risk of infectious disease from this degree of exposure is very low and limited to a few case reports. Strikingly, on a recent survey by Paterson, nearly 28% of urologists do not wear eye protection during ureteroscopy, while 40% wear laser goggles and 23% wear regular eyeglasses (39). It should be noted that this study was conducted in the pre-COVID era.

In summary, eye protection should be worn during ureteroscopy. The degree of eye protection varies depending on the case being performed. Plastic face-shields or glasses are appropriate for cystoscopy and holmium laser usage. However, when using lasers such as the diode, KTP, NG:YAG specialized glasses to cover that laser's wavelength should be considered. For high volume endourologists using extensive fluoroscopy for >20 cases per month, lead glasses should be considered to decrease the risk of long-term cataract formation.

## Improving Surgical Ergonomics

Surgeons maintain prolonged static postures and place their body under various biomechanical stresses to operate. This stress leads to fatigue, discomfort, and in the worst cases, injury. Across all specialties roughly half of all surgeons will develop injuries significant enough to seek medical care, 1 in 3 will decrease case volume, and 1 in 5 will miss work due to an injury (40, 41). In a study by Elkoushy et al., 64% of endourologists reported orthopedic related discomfort, with greatest prevalence in endourologists 40 years of age and older who had practiced for >10 years. The most common complaints were back problems (38.1%), neck problems (27.6%), hand problems (17.2%), and hip/knee problems (14.2%) (42). These complaints occur at an increased rate compared to our non-procedural peers. Healy et al. found that 32% of endourologists had hand/wrist complaints compared with only 19% of psychiatrists. The authors also report that surgeons who used counterintuitive ureteroscope deflection were significantly more likely to have complaints (56%) compared with those who used intuitive deflection (27%) (43). Unfortunately, 1 in 10 urologists reports ultimately needing corrective surgery to address these issues (44). Past research has shown it takes ~5 years among workers with highly repetitive hand activities to develop problems like tendonitis (44). Awareness is key to preventing long term disabilities.

Certainly, more ergonomic platforms such as the Avicenna Roboflex™ would help with these issues but they are not currently widely available (45). However, use of various types of endoscopes and their effect on ergonomics has been researched. Ludwig et al. measured surgeon biomechanics via EMG placement on 7 different upper extremity muscle beds and compared the differences in muscle activation when using 3 different ureteroscopes: Lithovue, Flex-X^C^, Flex-X^2^. The most highly activated muscles were the thenar groups and the extensor carpi ulnaris; overuse of the latter can result in the common condition of tennis elbow. They reported that digital ureteroscopes resulted in less muscle activation and therefore less surgeon fatigue and better ergonomics, likely attributable to the decreased weight of these scopes (46).

Ultimately, surgeons have control over their own body positioning in addition to the position of the patient and can take steps to improve their own ergonomics (Table 2). It is important to be comfortable. In an excellent review, Gabrielson et al. details the ideal ergonomics during ureteroscopy. The display monitors should be positioned directly in front of the surgeon at eye level to allow for <30 degrees of neck angulation and at a distance of 80–120 cm away. The upper body should be in a neutral position, with elbows bent between 90 and 120 degrees with the arms abducted no more than 30 degrees (35). A finger grip of the ureteroscope is preferable to a palm grip (36). The surgeon should then primarily engage wrist and finger muscles to maneuver the scope and avoid large inefficient movements of the shoulders or elbows. Additionally, dorsiflexion during foot pedal use should be limited to <25 degrees (35). It is important to distribute weight evenly when using a foot pedal. Alternating feet throughout the case can ease stress as well. If lead aprons must be used >10 h per week then 2-piece lead aprons are recommended and have been shown to improve weight distribution (47).

**Table 2:** Ergonomic recommendations by body region (35, 36).

Head	Monitors should be placed at eye level directly in front of the surgeon
Upper body	Elbows should be bent between 90 and 120 degrees with the arms abducted no further than 30 degrees away from the body
Hands	Finger grip of ureteroscope is preferrable to a “palm” grip with the wrist and finger muscles primarily used to maneuver the scope
Lower body	Distribute weight evenly and dorsiflex no >25 degrees when using foot pedals

## Limitations

There are several limitations of this review. First, adverse events are known to be underreported due to the voluntary nature of reporting thus it is difficult to characterize the true magnitude of the risks posed by these hazards. Second, many of the studies cited were conducted at a single institution thus, their results may not be generalizable due to unique practice environments. Finally, studies seeking to assess and quantify the degree of orthopedic and other issues that arise from posture issues are subjective, survey based, and thus inherently fail to capture the entire cohort that authors seek to characterize. Despite these limitations we sought to describe several known work-place hazards for the urologist, the degree of potential risk of these hazards, and provide easy to implement solutions to mitigate these hazards.

## Conclusion

Urologists are at risk for occupational radiation exposure and bodily injury. Procedures to mitigate this risk should be undertaken at all times including donning of lead aprons, eye protection, and maintaining ergonomic posture. In addition, keeping radiation use as low as reasonably achievable “ALARA” improves safety not only for the urologist but also for our patients and support staff.

## Author Contributions

DM and MS: manuscript writing and literature review. All authors contributed to the article and approved the submitted version.

## Conflict of Interest

The authors declare that the research was conducted in the absence of any commercial or financial relationships that could be construed as a potential conflict of interest.

## Publisher's Note

All claims expressed in this article are solely those of the authors and do not necessarily represent those of their affiliated organizations, or those of the publisher, the editors and the reviewers. Any product that may be evaluated in this article, or claim that may be made by its manufacturer, is not guaranteed or endorsed by the publisher.
